# Newly identified oncolytic VSV-GP-specific CD8^+^ T cell epitopes for monitoring of anti-viral immune responses in the BALB/c mouse model

**DOI:** 10.1016/j.omton.2025.201072

**Published:** 2025-10-24

**Authors:** Sarah Danklmaier, Saskia V. Vijver, Lisa Pipperger, Gabriel Floriani, Lukas Perro, Vanessa Konrad, Tamara Hofer, Hubert Hackl, Krishna Das, Guido Wollmann

**Affiliations:** 1 Institute of Virology, Medical University of Innsbruck, 6020 Innsbruck, Austria; 2 Christian Doppler Laboratory for Viral Immunotherapy of Cancer, Medical University of Innsbruck, 6020 Innsbruck, Austria; 3 Tyrolean Cancer Research Institute (TKFI), 6020 Innsbruck, Austria; 4 Department of Internal Medicine V, Haematology & Oncology, Medical University of Innsbruck, 6020 Innsbruck, Austria; 5 Institute of Bioinformatics, Biocenter, Medical University of Innsbruck, 6020 Innsbruck, Austria; 6 ViraTherapeutics GmbH, 6063 Rum, Austria

**Keywords:** MT: Regular Issue, VSV, CD8^+^ T cell epitope, prediction, oncolytic virus, anti-viral immunity, MHC-I, CT26 tumor model, BALB/c

## Abstract

The vesicular stomatitis virus variant VSV-GP is an oncolytic virus (OV) platform extensively studied in preclinical settings, which recently entered clinical trial testing. For oncolytic virotherapy, innate and adaptive immune system activation are considered major contributors. However, upon OV treatment, in addition to potential anti-tumor, anti-viral T cells are also raised, and comprehensively monitoring these anti-viral T cells presents a major challenge. Therefore, we aimed to identify anti-viral CD8^+^ T cells upon VSV-GP treatment in the widely utilized BALB/c mouse model using a multi-level adapted bioinformatics viral epitope prediction approach. Predicted viral epitopes presented on BALB/c major histocompatibility complex class I (MHC-I) alleles H2-Kd, H2-Dd, and H2-Ld were validated using ELISpot assay and intracellular cytokine staining. Subsequently, custom peptide-MHC-I multimers generated with the newly identified epitopes were used to directly detect virus-specific CD8^+^ T cells. Additionally, anti-viral CD8^+^ T cell dynamics of different treatment routes and multivirus exposure status in CT26.CL25 tumors and the spleen were analyzed. Taken together, the 11 newly identified epitopes facilitate the monitoring of anti-viral CD8^+^ T cells, which will aid the preclinical development of novel VSV-GP variants. This epitope-specific monitoring also serves as proof of concept for the potential future application of anti-viral immunomonitoring in clinical trial settings.

## Graphical abstract

**Figure:**
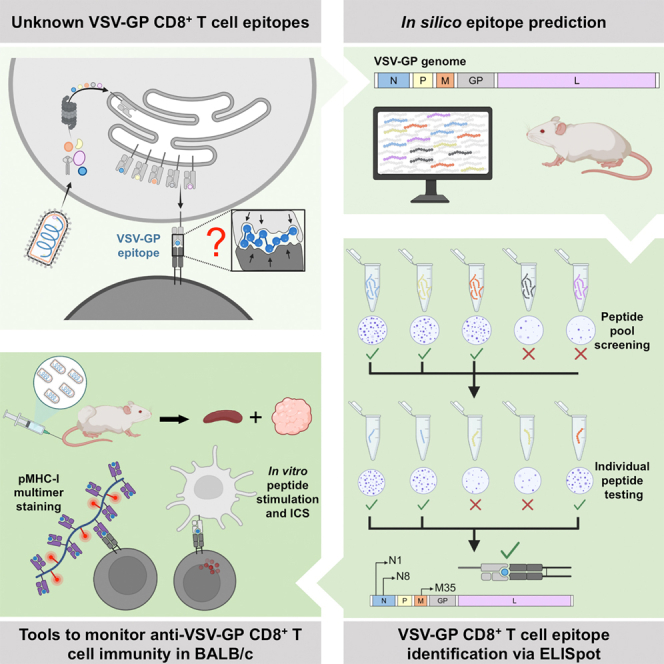

## Teaser

Wollmann and colleagues identified VSV-GP-specific CD8^+^ T cell epitopes in the widely utilized BALB/c mouse strain by using a multi-level adapted bioinformatics epitope prediction approach in combination with immune assays. These identified epitopes enable the monitoring of virus-specific CD8^+^ T cell dynamics of different treatment routes in tumor-bearing mice.

## Introduction

Oncolytic virus (OV) therapy is based on naturally occurring or engineered viruses that preferentially infect and kill cancer cells while leaving healthy cells unharmed.^1^ VSV-GP, a genetically engineered vesicular stomatitis virus (VSV) that encodes a foreign glycoprotein (GP) from the lymphocytic choriomeningitis virus (LCMV) instead of the wild-type VSV-GP, has shown promising oncolytic activity in various preclinical models.^2^^,^^3^^,^^4^^,^^5^ Displaying a rapid replication cycle, a broad tumor cell tropism, as well as a lack of pre-existing immunity in humans makes it a suitable OV candidate for local and systemic application.^2^ Additionally, VSV-GP showed promising preclinical results as a cancer vaccine vector in combination with vaccine partners in various mouse models.^6^^,^^7^

In part due to their origin as microbial agents, OVs possess the potential to turn immune cell-excluded tumors into highly immune cell-infiltrated tumors. This inflamed tumor microenvironment can sensitize tumors to immune checkpoint blockade (ICB) treatment, thus overcoming ICB resistance.^8^ VSV-GP treatment also leads to high intratumoral CD8^+^ T cell infiltration and immune cell activation in susceptible tumor models. However, some of our previous studies revealed that a substantial proportion of identified CD8^+^ T cells are of anti-viral origin.^5^^,^^6^^,^^9^ Numerous preclinical investigations have demonstrated that anti-viral T cells can exert dual effects on oncolytic virotherapy. For instance, these anti-viral T cells can impair the anti-tumor CD8^+^ T cell responses by limiting viral replication and spread.^10^ On the other hand, anti-viral CD8^+^ T cells may also positively impact the therapeutic effect via indirect mechanisms.^11^ Upon viral infection, viral peptides are degraded intracellularly, with a fraction of them presented as epitopes on the cell surface by major histocompatibility complex (MHC) molecules, including MHC class I (MHC-I). These peptide-MHC-I (pMHC-I) complexes can be recognized by virus-specific CD8^+^ T cells through their highly specific T cell receptor (TCR), thereby initiating activation and clonal expansion of CD8^+^ T cells.^12^ Due to high polymorphisms concentrated in peptide-binding grooves of different MHC alleles, a great diversity of peptides can bind in an allele-specific manner.^13^ Bioinformatics prediction tools, based on machine learning algorithms that are trained with experimental data on peptide binding affinity, offer a valuable approach to predict potential immunogenic T cell candidate epitopes.^14^ This computational methodology can be applied for the prediction of viral T cell epitopes, e.g., of hepatitis C virus and also works for the identification of neoepitopes in cancer.^15^^,^^16^^,^^17^

Anti-viral CD8^+^ T cell dynamics have often been underexplored in preclinical and clinical studies, even though their influence on the efficacy of OV therapy is ambiguous.^11^ In the present study, we therefore sought to identify anti-viral CD8^+^ T cells upon VSV-GP treatment in the widely utilized BALB/c mouse strain. This preclinical mouse model expresses H2-Kd, H2-Dd, and H2-Ld MHC-I alleles.^18^ Employing an adapted bioinformatics epitope prediction approach, viral epitopes for the BALB/c alleles were predicted. By screening these predicted peptide pools using VSV-GP-immunized mice, individual immunogenic epitopes were identified and validated using ELISpot assay, intracellular cytokine staining (ICS), and MHC-multimer staining. Moreover, these newly identified epitopes allowed the in-depth monitoring of anti-viral CD8^+^ T cells upon VSV-GP treatment in the CT26.CL25 colon carcinoma tumor model.

## Results

### Pool screening identifies nine H2-Kd-, six H2-Dd-, and four H2-Ld-activating peptide pools

**Figure 1:**
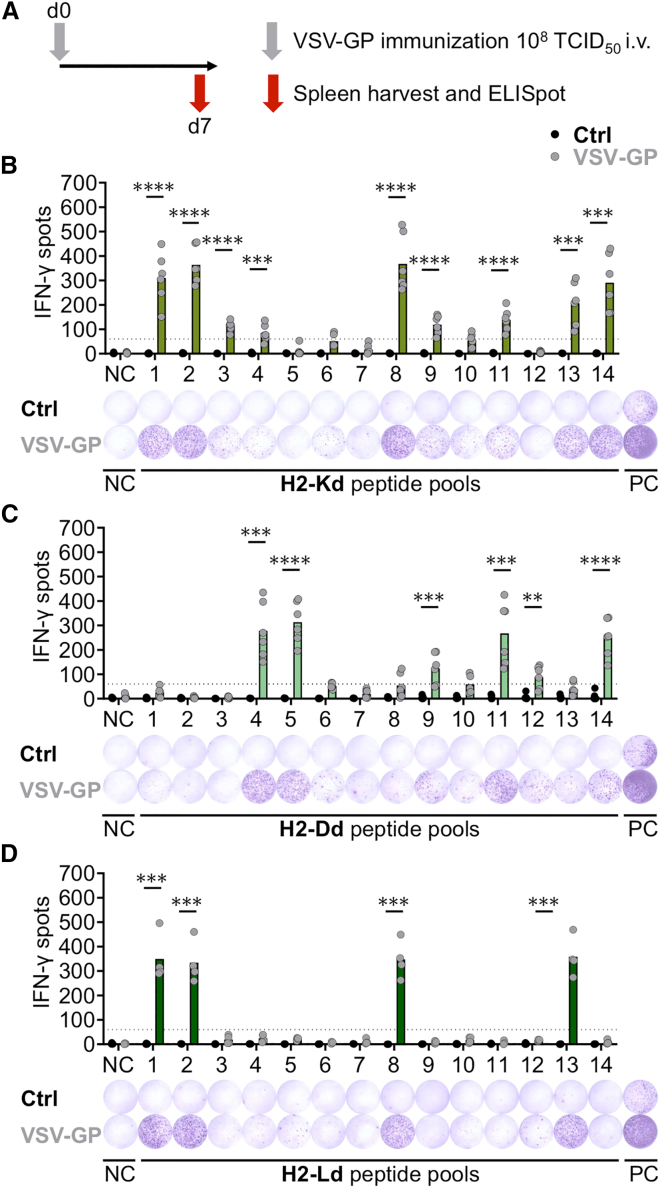
IFN-γ ELISpot assays identify activating H2-Kd, H2-Dd, and H2-Ld peptide pools in VSV-GP-immunized BALB/c mice (A) Schematic schedule of the experimental setup is shown. BALB/c mice received 10^8^ TCID_50_ VSV-GP i.v. on day 0. Seven days later, spleens of untreated control (Ctrl) and VSV-GP-treated (VSV-GP) mice were used for IFN-γ ELISpot assays. (B–D) IFN-γ spot counts using 2.5 × 10^5^ splenocytes per well stimulated with 35 μg/mL H2-Kd, H2-Dd, and H2-Ld peptide pools 1–14 are depicted. Additionally, representative IFN-γ ELISpot images are shown. Unstimulated cells and ConA (5 μg/mL) were used as negative control (NC) and positive control (PC), respectively. Data from three (H2-Kd and H2-Dd) or two (H2-Ld) independent experiments are depicted (*n* = 6 for H2-Kd and H2-Dd and *n* = 4 for H2-Ld). IFN-γ secretion of samples from VSV-GP-immunized mice is compared to samples of untreated mice, and statistically significant differences are indicated with asterisks (unpaired *t* test). ∗∗*p* ≤ 0.01; ∗∗∗*p* ≤ 0.001; ∗∗∗∗*p* ≤ 0.0001. The black dotted line indicates the minimum spot count (60 counts) for the analysis to be considered acceptable. Bars indicate the mean spot count.

To assess the full range of the anti-viral CD8^+^ T cell response upon VSV-GP treatment in BALB/c mice, we first performed epitope predictions based on the amino acid sequence of VSV-GP. The workflow of epitope prediction and selection was described previously by our lab and has been used to identify previously unknown VSV-GP-specific epitopes for the MHC-I alleles H2-Db and H2-Kb.^17^ In the current study, we aimed to predict H2-Kd-, H2-Dd-, and H2-Ld-presented epitopes. Around 1,000 epitopes per MHC-I allele were predicted, and the highest-ranked epitopes (50 epitopes per MHC-I allele) were selected and included in a peptide matrix for further validation. For each MHC-I allele, 14 peptide pools were screened to identify those that could activate T cells. BALB/c mice were immunized intravenously (i.v.) with VSV-GP at a dose of 10^8^ TCID_50_. Seven days later, splenocytes were harvested and stimulated *ex vivo* with peptide pools in an interferon (IFN)-γ ELISpot assay, which measures IFN-γ secretion as a marker of T cell activation (Figure 1A). Splenocytes of VSV-GP-immunized BALB/c mice responded to several peptide pools from all three MHC-I alleles with varying IFN-γ spot counts. Overall, nine H2-Kd (Figure 1B), six H2-Dd (Figure 1C), and four H2-Ld (Figure 1D) peptide pools induced a significant number of IFN-γ spot counts in VSV-GP-treated (VSV-GP) compared to untreated mice (Ctrl). Exemplary IFN-γ spot images are depicted below each respective graph (Figures 1B–1D). To identify individual immunogenic peptide candidates, a matrix deconvolution was performed, in which non-activating pools were crossed out and only individual candidates of activating peptide pools were further evaluated (Figure S1).

### Individual peptide testing identifies five H2-Kd-, four H2-Dd-, and two H2-Ld-presented VSV-GP epitopes

**Figure 2:**
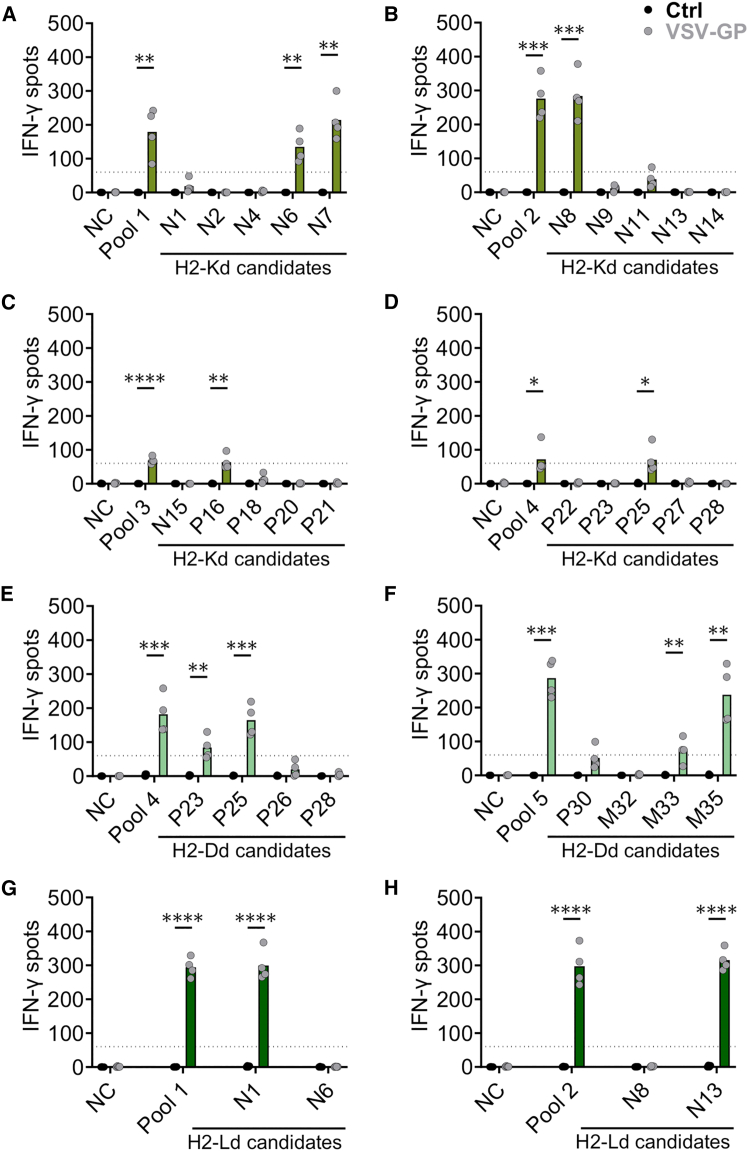
Identification of H2-Kd-, H2-Dd-, and H2-Ld-presented VSV-GP-specific CD8^+^ T cell epitopes in BALB/c mice using IFN-γ ELISpot assays BALB/c mice were immunized with VSV-GP (10^8^ TCID_50_, i.v.). Seven days later, splenocytes of untreated (Ctrl) and VSV-GP-treated (VSV-GP) mice were isolated and used for ELISpot assays. Splenocytes (2.5 × 10^5^ cells/well) were stimulated with 35 μg/mL peptide pool or 5 μg/mL individual peptides. Unstimulated cells were used as negative control (NC). IFN-γ spots induced by H2-Kd (A–D), H2-Dd (E and F), and H2-Ld (G and H) candidates with their respective activating vertical peptide pools are depicted. Data from two independent experiments are shown (*n* = 4). IFN-γ secretion of samples from VSV-GP-immunized mice is compared to untreated mice, and statistically significant differences are indicated with asterisks (unpaired *t* test). ∗*p* < 0.05; ∗∗*p* ≤ 0.01; ∗∗∗*p* ≤ 0.001; ∗∗∗∗*p* ≤ 0.0001. The black dotted line indicates the minimum spot count (60 counts) for the analysis to be considered acceptable. Bars indicate the mean spot count.

To identify individual immunogenic epitopes, single peptides of activating pools were further screened in IFN-γ ELISpot assays. Splenocytes of untreated and VSV-GP-treated mice were *ex vivo* stimulated with the respective peptides and were analyzed for IFN-γ release 7 days after the virus immunization. Overall, 20 H2-Kd, eight H2-Dd, and four H2-Ld peptides together with their respective activating vertical peptide pools were tested (Figure 2). From the screened peptides, five H2-Kd- (Figures 2A–2D), four H2-Dd- (Figures 2E and 2F), and two H2-Ld-presented peptides (Figures 2G and 2H) induced statistically significant IFN-γ spot counts. Representative IFN-γ ELISpot images are provided in Figures S2A–S2F. Specifically, for the H2-Kd allele, three VSV-N epitopes, N6, N7 (part of pool 1), and N8 (part of pool 2), and two VSV-P epitopes, P16 (part of pool 3) and P25 (part of pool 4), were identified. Reactive H2-Dd-presented epitopes consist of two VSV-P epitopes, P23 and P25 (part of pool 4), and two VSV-M epitopes, M33 and M35 (part of pool 5). Lastly, two H2-Ld epitopes were identified both from the VSV-N protein, namely N1 (part of pool 1) and N13 (part of pool 2). Even though the number of identified epitopes for the H2-Kd and H2-Dd allele was higher compared to the H2-Ld allele (five, four, and two identified epitopes, respectively), the two identified H2-Ld VSV-N peptides induced a marginally higher number of spots, although not statistically significant (Figure S2G).

### ICS validates the identified VSV-GP-specific CD8^+^ T cell epitopes

**Figure 3:**
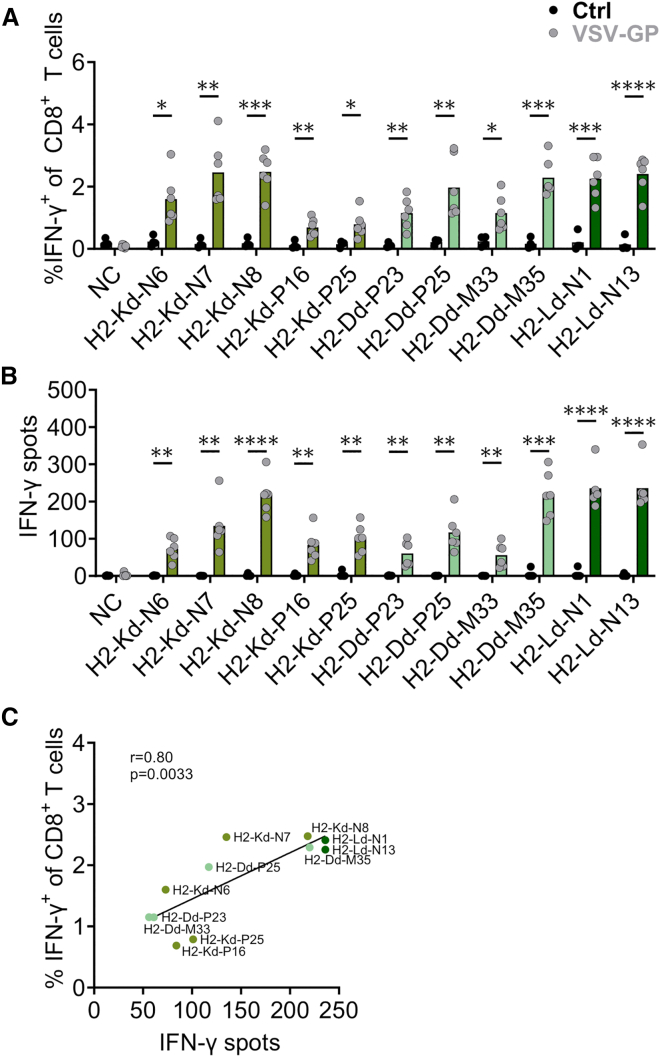
Intracellular IFN-γ staining confirms the immunogenicity of newly identified H2-Kd-, H2-Dd-, and H2-Ld-presented epitopes Splenocytes of untreated (Ctrl) and VSV-GP-immunized (VSV-GP, 10^8^ TCID_50_, i.v.) BALB/c mice were restimulated with either 10 μg/mL or 5 μg/mL epitopes, and the cells were used for intracellular IFN-γ staining (7.5 × 10^5^ cells/well) or IFN-γ ELISpot (2.5 × 10^5^ cells/well), respectively. Unstimulated cells were used as negative control (NC). (A) The frequencies of IFN-γ^+^ cells among CD8^+^ T cells following ICS are shown. (B) IFN-γ spot counts following ELISpot assay are depicted. (C) The correlation of mean values of IFN-γ spot counts in the ELISpot assay and the frequencies of IFN-γ^+^ among CD8^+^ T cells in the ICS staining are shown. Data are derived from two independent experiments (*n* = 4 for untreated, *n* = 6 for VSV-GP). Intracellular IFN-γ production and IFN-γ spot counts of VSV-GP-immunized mice are compared to untreated mice, and statistically significant differences are indicated with asterisks (unpaired *t* test). ∗*p* < 0.05; ∗∗*p* ≤ 0.01; ∗∗∗*p* ≤ 0.001; ∗∗∗∗*p* ≤ 0.0001. For correlation, the Pearson correlation coefficient was used.

To further corroborate the immunogenicity of the individual peptides, splenocytes of untreated and VSV-GP-treated mice were *ex vivo* stimulated with H2-Kd-, H2-Dd-, and H2-Ld-presented peptides and analyzed for intracellular IFN-γ production using ICS followed by flow cytometric analysis. The fluorescence-activated cell sorting (FACS) gating strategy and exemplary positive as well as negative control samples are depicted in Figure S3A. Consequently, we assessed the frequencies of IFN-γ^+^ CD8^+^ T cells (Figure 3A). The immunogenicity of individual peptides was validated for all 11 viral epitopes by a statistically significant increase of IFN-γ frequencies compared to controls. In parallel, corresponding IFN-γ ELISpots were repeated (Figure 3B), confirming the results from Figure 2. IFN-γ signals derived from the ICS staining correlated strongly with IFN-γ spot counts from corresponding ELISpots (Figure 3C). Of note, the predicted MHC-I-presented peptides did not stimulate CD4^+^ T cells, as no IFN-γ^+^ signal among CD4^+^ T cells was detected in the ICS experiments (Figure S3B).

### Characteristics of the identified VSV-GP-specific CD8^+^ T cell epitopes

**Figure 4:**
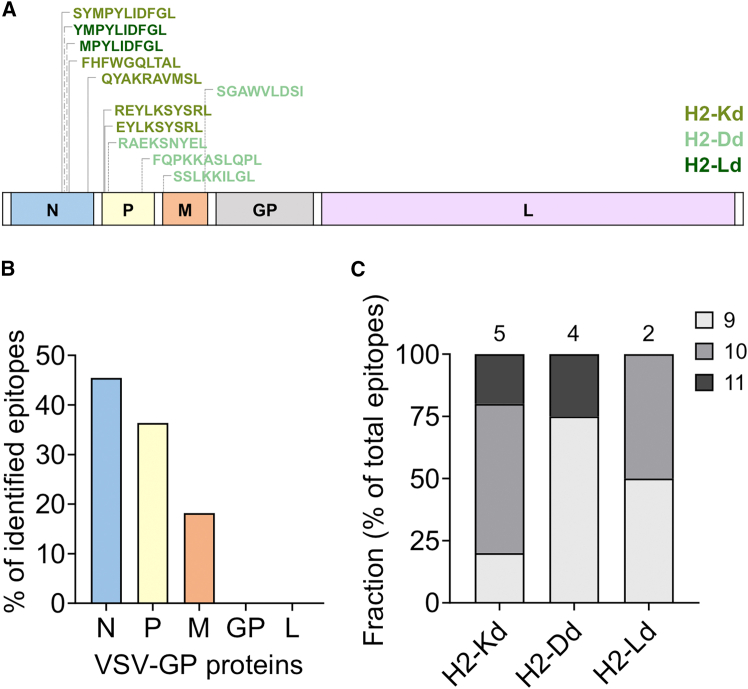
VSV-GP-specific CD8^+^ T cell epitopes are distributed across the first three viral proteins and range from nine to 11 amino acids (A) Location of H2-Kd-, H2-Dd-, and H2-Ld-presented epitopes in the viral proteome. The epitope sequences are indicated in different colors for each MHC-I allele. (B) Percentages of total identified epitopes per viral protein combined across all three MHC-I alleles are shown. (C) Relative distribution of the peptide length in amino acids (9, 10, or 11 amino acids) for H2-Kd-, H2-Dd-, and H2-Ld-presented epitopes. The numbers above the bars indicate the total number of identified epitopes per MHC-I allele.

Overall, five H2-Kd- (N6, N7, N8, P16, and P25), four H2-Dd- (P23, P25, M33, and M35), and two H2-Ld-presented (N1 and N13) VSV-GP-specific CD8^+^ T cell epitopes were identified. Interestingly, the predicted epitope’s binding affinity to corresponding MHC alleles was quite low for several epitopes (H2-Kd-N7, H2-Kd-N8, H2-Dd-P23, H2-Dd-P25, H2-Dd-M33, and H2-Dd-M35). Nevertheless, these epitopes still induced significant IFN-γ levels in both immune assays, indicating that additional factors apart from the MHC binding affinity contribute to the immunogenicity of peptides (Table S1). The distribution of the immunogenic epitopes within the viral proteome is presented in Figure 4A. All 11 epitopes are spread within the first three proteins of VSV-GP. Specifically, the VSV-N-protein harbors five epitopes, while the VSV-P-protein and VSV-M-protein encode four epitopes and two epitopes, respectively (Figure 4B). No activating epitopes were identified for the LCMV-GP and the VSV-L protein in the BALB/c mouse strain. Collectively, the peptide length of the identified epitopes varied from nine to 11 amino acids, with 9-mers being the predominant epitope length (Figure 4C).

### Effect of immunization route on the frequency and composition of VSV-GP-specific CD8^+^ T cells in the tumor and spleen

**Figure 5:**
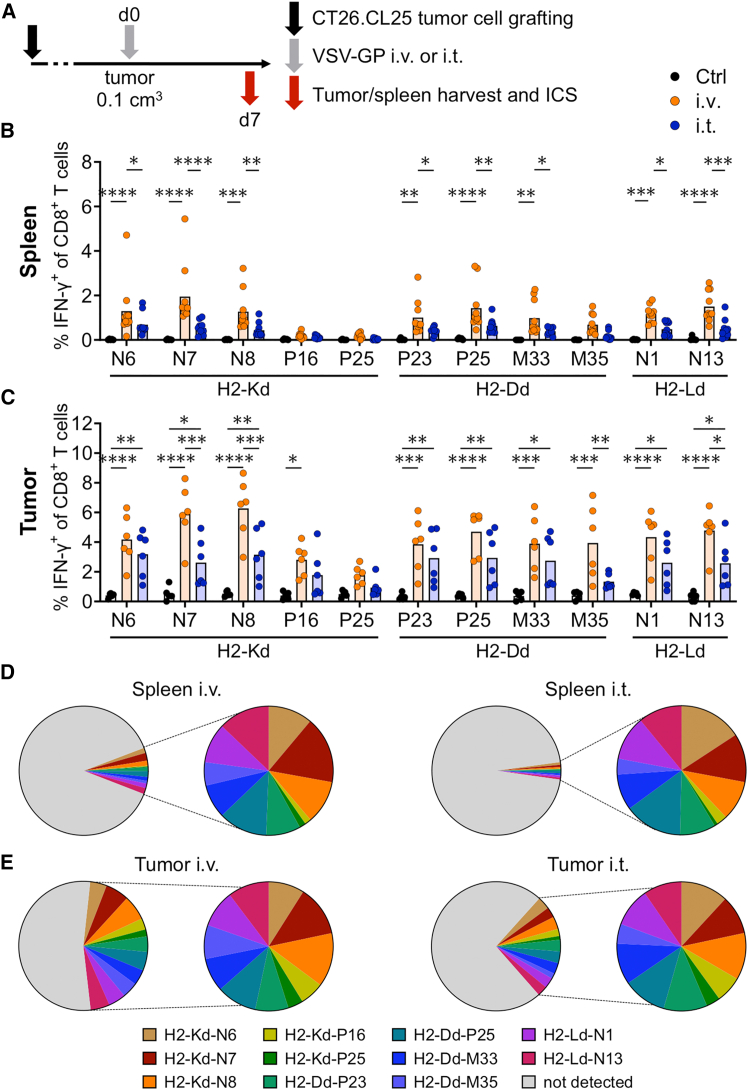
Intravenous VSV-GP treatment increases the induction of anti-viral IFN-γ^+^ CD8^+^ T cells compared to intratumoral application (A) Schematic schedule of the experimental setup. BALB/c mice were injected s.c. with colon carcinoma CT26.CL25 cells (10^5^/per mouse). At a tumor size of around 0.1 cm^3^, mice were immunized with 10^8^ TCID_50_ VSV-GP either i.v. or i.t. Seven days later, tumors and spleens of untreated (Ctrl) and VSV-GP-immunized (VSV-GP) BALB/c mice were harvested for intracellular IFN-γ staining. (B and C) Percentages of intracellular IFN-γ^+^ CD8^+^ T cells in response to H2-Kd, H2-Dd, and H2-Ld VSV-GP peptide stimulation (10 μg/mL) measured in the splenocytes and TILs. (D and E) The left pie charts depict the mean values of IFN-γ frequencies among all CD8^+^ T cells in the splenocytes or the TILs after i.v. and i.t. treatment. The right pie charts show the relative proportion of the identified virus-specific CD8^+^ T cells against the indicated epitopes. Data from two independent experiments are shown (tumor *n* = 5 for untreated and *n* = 6 for VSV-GP; spleen *n* = 5 for untreated and *n* = 10 for VSV-GP). IFN-γ production from samples of VSV-GP-immunized mice is compared to samples of untreated mice, and statistically significant differences are indicated with asterisks (two-way ANOVA with Tukey’s multiple comparison). ∗*p* < 0.05; ∗∗*p* ≤ 0.01; ∗∗∗*p* ≤ 0.001; ∗∗∗∗*p* ≤ 0.0001.

Next, the impact of the most commonly used virotherapy application routes—i.v. or intratumoral (i.t.) application^19^—on the frequency and diversity of the anti-viral immune response was evaluated. For this, VSV-GP-specific CD8^+^ T cell responses were monitored using the identified VSV-GP BALB/c epitopes in mice engrafted with the widely used colon carcinoma cell line CT26.CL25 and treated with 10^8^ TCID_50_ VSV-GP either i.v. or i.t. Seven days post OV treatment, spleens and tumors were harvested; splenocytes and tumor-infiltrating lymphocytes (TILs) were stimulated *ex vivo* with the 11 immunogenic H2-Kd-, H2-Dd-, and H2-Ld-presented peptides and were analyzed for their ability to induce intracellular IFN-γ production in T cells (Figure 5A). When comparing the VSV-GP-specific CD8^+^ T cell response of splenocytes based on the treatment route, eight out of 11 peptides induced statistically significant higher IFN-γ frequencies after i.v. compared to i.t. treatment (Figure 5B). Specifically, the Kd-presented peptides H2-Kd-N6, H2-Kd-N7, and H2-Kd-N8 generated significantly elevated intracellular IFN-γ^+^ frequencies in splenocytes in i.v.- compared to i.t.-treated mice. Moreover, all Dd- and Ld-presented peptides except H2-Dd-M35 induced higher proportions of IFN-γ^+^ CD8^+^ splenocytes in i.v.- compared to i.t.-treated animals. Regarding the IFN-γ frequencies among CD8^+^ T cells in TILs, we observed similar tendencies as in splenocytes, even though overall frequencies were higher in TILs compared to the splenocytes (Figure 5C). Mean values of IFN-γ frequencies among CD8^+^ T cells after VSV-GP treatment reached up to 46.44% (i.v.) and 26.80% (i.t.) in the TILs and 11.65% (i.v.) and 4.35% (i.t.) in splenocytes. Overall, the distribution pattern of IFN-γ levels upon stimulation with the 11 peptides was highly comparable in the tumor and spleen upon i.v. and i.t. treatment (Figures 5D and 5E). In addition, a polyfunctionality assay was performed for three newly identified peptides: H2-Kd-N7, H2-Dd-P25, and H2-Ld-N1 (Figure S4). None of the three anti-viral CD8^+^ T cell specificities displayed triple-positive TNF-α^+^IFN-γ^+^CD107a^+^ CD8^+^ T cells upon peptide restimulation of splenocytes. However, single-positive IFN-γ and CD107a-producing cells as well as double-positive IFN-γ^+^CD107a^+^ CD8^+^ T cells were detected, indicating successful degranulation of IFN-γ-containing vesicles upon antigen recognition. The dynamics comparing the two treatment routes were consistent for CD107a expression and IFN-γ^+^CD107a^+^ double-positive cells, with i.v. showing higher frequencies than i.t. Furthermore, an additional experiment addressed whether the presence of an existing tumor affects the VSV-GP-specific CD8^+^ T cell immunity after i.v. virus treatment in terms of magnitude and epitope distribution. Our results suggest that the presence of CT26.CL25 tumors does not influence the virus-specific T cell response as neither changes in IFN-γ^+^ CD8^+^ T cell frequencies and total counts nor in the epitope distribution were observed between tumor-bearing and non-tumor-bearing mice (Figures S5A–S5F). Last, we examined whether the presence of pre-existing VSV-GP-specific CD8^+^ T cells had an impact on anti-viral CD8^+^ T cell frequencies. The experimental schedule as in Figure 5A was applied with two groups of mice being pre-immunized with VSV-GP i.v. 3 weeks prior to tumor implantation. As shown in Figure S5G, pre-immunization did not alter IFN-γ^+^ CD8^+^ T cell frequencies following restimulation with either H2-Kd-N7, H2-Dd-P25, or H2-Ld-N1 peptides.

### Custom-made pMHC-I multimers of newly identified VSV-GP epitopes detect virus-specific CD8^+^ T cells

**Figure 6:**
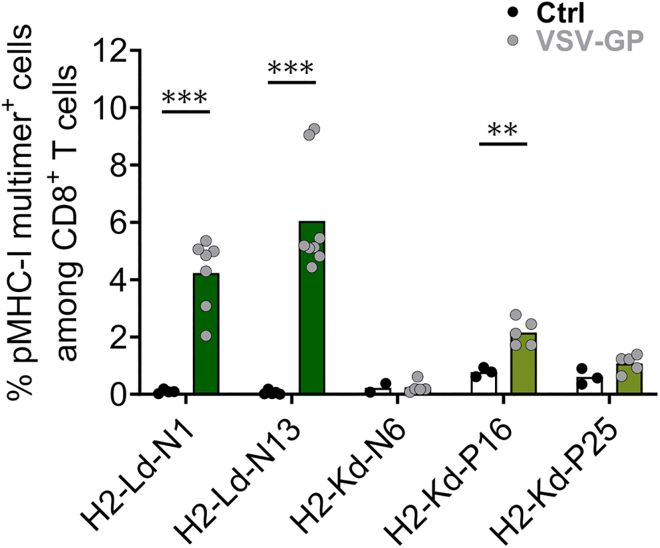
Custom-made pMHC-I multimers detect several VSV-GP-specific CD8^+^ T cell epitopes A dose of 10^8^ TCID_50_ VSV-GP was injected i.v. into BALB/c mice. After seven days, spleens of untreated (Ctrl) and VSV-GP-immunized (VSV-GP) mice were harvested for pMHC multimer staining measured via flow cytometry. Percentages of H2-Ld-N1, H2-Ld-N13, H2-Kd-N6, H2-Kd-P16, and H2-Kd-P25 multimer^+^ CD8^+^ T cells are shown. Data are derived from three independent experiments (*n* = 3–5 for untreated and *n* = 5–8 for VSV-GP). Frequencies of multimer^+^ CD8^+^ T cells of VSV-GP-immunized mice are compared to untreated mice, and statistically significant differences are indicated with asterisks (unpaired *t* test). ∗∗*p* ≤ 0.01; ∗∗∗*p* ≤ 0.001.

Using pMHC-I multimers, virus-specific CD8^+^ T cells can be labeled directly without *ex vivo* peptide restimulation. Based on their MHC binding affinities, five custom-made pMHC-I multimers were generated to measure VSV-GP-specific CD8^+^ T cells, namely H2-Kd-N6, H2-Kd-P16, H2-Kd-P25, H2-Ld-N1, and H2-Ld-N13. As the pMHC-I affinity varies between different epitopes, not all epitopes were suitable for the production of pMHC-I multimers (Table S1). No H2-Dd candidate was selected, as the NetMHC-4.0 tool predicted affinity values above 1,000 nM, which is over the cutoff value set by the manufacturer for pMHC-I multimers to be produced. Splenocytes of untreated and VSV-GP-treated mice were stained with pMHC-I multimers and analyzed by flow cytometry 7 days after i.v. immunization with 10^8^ TCID_50_ VSV-GP. The percentages of H2-Ld-N1, H2-Ld-N13, H2-Kd-N6, H2-Kd-P16, and H2-Kd-P25 multimer^+^ CD8^+^ T cells are shown in Figure 6. While H2-Ld-N1, H2-Ld-N13, and H2-Kd-P16 pMHC-I multimers identified clearly distinguishable pMHC-I multimer^+^ populations, H2-Kd-N6 and H2-Kd-P25 multimers either showed no or only low frequencies of labeled CD8^+^ T cells. These small populations of pMHC-I multimer^+^ CD8^+^ T cells for the last three epitopes do not match the high IFN-γ^+^ signals detected in the ICS and ELISpot indicating discrepancies between the different assays.

## Discussion

In the past decades of preclinical and clinical oncolytic virotherapy studies, monitoring of virus-specific CD8^+^ T cells has largely been unexplored, mostly due to technical limitations and unknown identity of virus-specific T cell epitopes for many OVs. However, detecting, quantifying, and understanding the functionality of anti-viral T cell responses may help to evaluate and optimize oncolytic virotherapeutic efficacy. Comprehensive monitoring of VSV-GP-specific CD8^+^ T cell responses in the widely used preclinical BALB/c mouse model has been elusive so far as the VSV-specific epitope characterization was largely unknown.

Hence, we utilized an adapted bioinformatics epitope prediction approach in combination with immune assays to predict and identify immunogenic VSV-GP-specific CD8^+^ T cell epitopes similar to our previous study to identify MHC-I epitopes of VSV-GP in another preclinical model, the C57BL/6J mice.^17^ Moreover, the same bioinformatics tools have proven successful in identifying neoepitopes.^20^^,^^21^ Additionally, after the outbreak of SARS-CoV-2, these epitope prediction algorithms identified SARS-CoV-2-specific CD8^+^ T cells.^22^^,^^23^ However, *in silico* predicted epitopes need experimental validation to confirm their immunogenicity.

In this current study, we identified a total of 11 viral epitopes presented on mouse BALB/c MHC-I alleles H2-Kd, H2-Ld, and H2-Dd using the IFN-γ ELISpot assay-based multi-level adapted bioinformatics epitope prediction approach. We further validated our identified epitopes for their ability to induce IFN-γ production via ICS and found a strong correlation between IFN-γ ELISpot and ICS data. While ELISpots are highly sensitive assays, it is important to note that not only CD8^+^ T cells but also other immune cells can secrete IFN-γ. As such, IFN-γ ELISpot assay alone cannot confirm that the IFN-γ production originates solely from CD8^+^ T cells. ICS provides the advantage of cell subtype analysis alongside cytokine production.^24^

In general, MHC-I molecules present epitopes with a length of eight to 11 amino acids.^25^ Due to the highly polymorphic state of the MHC-I gene, various allelic variations of the MHC-I molecule exist.^12^ Some amino acid side chains of the peptide are buried into the six binding pockets, termed A–F, within the binding cleft of the MHC-I complex.^26^ The cleft architecture can differ between various MHC-I protein variants, shown by the absence of pocket E and pocket C in the H2-Kd and H2-Ld groove, respectively.^27^^,^^28^ For mouse MHC-I-presented epitopes, the C termini consist mostly of hydrophobic amino acids, as the mouse TAP protein prefers peptides with hydrophobic C-terminal ends.^29^ All of our identified epitopes harbor a hydrophobic C-terminal residue, consisting of either leucine or isoleucine. Taking the H2-Kd-presented epitopes, the amino acid sequences of the anchor residues are in line with the peptide-binding motifs found in the literature. The dominant anchor residues for H2-Kd-presented epitopes are tyrosine for position two and leucine or isoleucine for the C terminus.^30^ In accordance with these findings, the epitopes we identified predominantly display a tyrosine at position two, which interacts with the MHC-I binding pocket B. Due to the chemical nature, size, and shape of pocket B, tyrosine is strongly favored at this position. The C-terminal leucine, which as stated earlier we found for all identified H2-Kd-presented epitopes, is buried into pocket F.^27^ Lastly, pocket C allows the interaction of several amino acids.^27^^,^^31^ This variability of amino acids at position five is also present in our identified epitopes. The peptides presented by the H2-Dd allele are characterized by a four-residue binding motif, in which amino acid residues at positions two, three, five, and ten are almost completely buried into the binding groove.^32^ We identified epitopes with glycine at position two, proline at position three, lysine at position five, and leucine or isoleucine at the C terminus. These anchor residues are in line with H2-Dd-presented epitopes found in the literature.^29^^,^^32^ Regarding the H2-Ld allele, the binding cleft is more hydrophobic compared to other MHC-I molecules. This hydrophobicity of binding pocket B favors a proline as anchor residue, fitting our identified H2-Ld epitopes.^28^ In regard to sequence similarity between the newly identified VSV-GP-specific CD8^+^ T cell epitopes, H2-Ld-N1 (MPYLIDFGL) and H2-Ld-N13 (YMPYLIDFGL) as well as H2-Kd-P16 (EYLKSYSRL) and H2-Kd-P25 (REYLKSYSRL) differ by only one additional amino acid. Notably, this additional amino acid decreases the predicted affinity to the respective MHC-I molecule by almost 35-fold and 6-fold, respectively. However, all four epitopes are still defined as strong binders according to the NetMHC-4.0 server, and all induce comparable IFN-γ levels (Figure 3), indicating that the lower pMHC-I binding affinity is still sufficient to trigger an adequate immune response. One of the herein identified epitopes (MPYLIDFGL; H2-Ld-N1) had previously been described and used to measure virus-specific CD8^+^ T cells following VSV therapies.^33^^,^^34^ To our knowledge, all other identified epitopes have not been published before. Notably, comparing the identified VSV-GP-specific epitopes for the BALB/c mouse strain with our previously determined epitopes for the C57BL/6J mouse strain,^17^ we found the H2-Dd-P23 (FQPKKASLQPL) and H2-Dd-P25 (RAEKSNYEL) epitopes to be shared between both mouse strains (designated as H2-Db-P18 and H2-Db-P21 epitopes, respectively, in our previous study). Such sharing of immunoprevalent epitopes between human leukocyte antigen (HLA) or MHC variants has been shown with other viral epitopes.^35^^,^^36^

In contrast to the epitope repertoire in C57BL/6J mice, the identified epitopes in the BALB/c mice that induced a CD8^+^ T cell response were exclusively found within the first three viral proteins (VSV-N, VSV-P, and VSV-M), with the VSV-N protein being the most immunogenic protein containing five epitopes, followed by the VSV-P and the VSV-M proteins containing four and two epitopes, respectively. This hierarchy of immunogenicity might in part reflect the protein abundance gradient often seen in negative-strand RNA viruses following the principle of sequential gene expression.^37^ The protein closest to the 3′ shows the highest protein expression level, and all following protein amounts decrease within increasing distance to the 3′ end.^38^ Furthermore, our findings are consistent with T cell-mediated responses of other negative-strand RNA viruses. Sakabe et al. showed that after Ebola virus infections, the strongest CD8^+^ T cell responses are directed against epitopes within the most upstream proteins with a decrease of immunogenicity toward the 5′ end.^39^ While this natural gradient was purposely built into our prediction pipeline, with a candidate gradient decreasing from 15 candidate epitopes for the most upstream N protein down to 5 candidate epitopes for the most downstream L protein, this imbalance might also have attributed to a bias toward identifying epitopes of more upstream genes. Such a bias might play a bigger role for the BALB/c mouse compared to the previously reported epitopes in the C57BL/6 mouse when taking into account the generally higher Th1-skewed immunogenicity of the C57BL/6 mouse strain compared to the lesser immunogenic BALB/c.^40^ Previous reports emphasize peptide-dose-dependent effects on T cell-dendritic cell (DC) dynamics. Henrickson et al. showed that only when a certain threshold of peptide amount is reached, a T cell response is triggered.^41^ Notably, our workflow identified in total 20 immunogenic VSV-GP-specific CD8^+^ T cell epitopes deriving from all proteins in the C57BL/6 mouse strain,^17^ compared to the 11 VSV-GP-specific CD8^+^ T cell epitopes in this study. Although a direct comparison was not performed, the identified VSV-GP-specific CD8^+^ T cell epitopes seem to be less immunogenic in the BALB/c compared to the C57BL/6 mouse strain, highlighted by overall lower IFN-γ^+^ frequencies. This is in line with a study comparing the CD8^+^ T cell immunity in these two mouse strains in response to SARS-CoV-2 infection. Virus-specific CD8^+^ T cell epitopes were found to be less frequent in the BALB/c mouse strain and also less immunogenic.^42^ This may suggest that C57BL/6 mice potentially mount a stronger CD8^+^ T cell response to viral infections compared to BALB/c mice. Another possible reason why overall fewer immunogenic epitopes were identified and validated in the BALB/c mouse strain compared to C57BL/6 might be due to the smaller number of training dataset used for BALB/c H2-Kd, H2-Ld, and H2-Dd alleles compared to the more extensively studied C57BL/6 H2-Db and H2-Kb alleles.^43^ The smaller dataset used to train NetMHCpan-4.1 could impact the precise prediction of MHC-I binding for BALB/c alleles, potentially affecting the proportion of experimentally validated immunogenic epitopes. However, differences in epitope distribution might also be attributable to some HLA allele-intrinsic factors. Shkurnikov et al. showed a distinct distribution pattern of SARS-CoV-2-derived CD8^+^ T cell epitopes in humans with different HLA types. While the majority of epitopes associated with the HLA-A∗01:01 allele originate from non-structural regions (ORF1ab), HLA-A∗07:02 epitopes derive from structural regions.^44^ This indicates that predominant epitope regions within viral genomes can differ from allele to allele.

As VSV-GP is an oncolytic agent developed for potential cancer therapies, we included the newly identified epitopes to comprehensively monitor VSV-GP-specific CD8^+^ T cells in virotherapy settings in tumor-bearing mice. Comparing different routes of administration, we found that the i.v. injections induced significantly higher intracellular IFN-γ^+^ CD8^+^ T cell frequencies for almost all peptides compared to i.t. treatment in the splenocytes of CT26.CL25 tumor-bearing mice. This expands on our previous reports on robust virus-specific CD8^+^ T cell responses observed in the periphery upon systemic VSV-GP application, although only a single viral epitope was included in those studies.^5^^,^^9^ Enhanced anti-viral responses upon systemic application may be linked to the role of CD169^+^ marginal zone macrophages (MZMs) in the clearance of blood-borne infections. MZMs capture blood-derived pathogens in the spleen and allow enforced viral replication of VSV as well as LCMV.^45^^,^^46^ Recently, this phenomenon has also been described for VSV-GP^47^ and could conceivably explain the robust induction of anti-viral T cell responses in the absence of a tumor. Unless a tumor shows high susceptibility for viral infection and propagation, the spleen might serve as a main antigen contributor for induction of an anti-viral CD8^+^ T cell response after systemic application of oncolytic VSV-GP. The i.t. treatment induced low but detectable systemic IFN-γ production in CD8^+^ T cells, which may be indicative of a leakage of virus particles into the bloodstream. Other studies supporting this argument found low levels of viral DNA in the spleen upon i.t. injections of OVs.^48^^,^^49^ A different explanation could be that migratory DCs transport virus antigens from the tumor to the tumor-draining lymph nodes to prime virus-specific CD8^+^ T cells, from where they can enter the blood circulation.^50^ Regarding the magnitude of IFN-γ production between epitopes, the same distribution pattern was observed in the tumor and spleen. However, the frequencies were increased in the tumor compared to the spleen, indicating that virus-specific CD8^+^ T cells concentrate at the site of viral infection. Furthermore, we showed that in the BALB/c model, the presence of a tumor does not influence VSV-GP-specific CD8^+^ T cells, as intracellular IFN-γ^+^ CD8^+^ T cell frequencies in splenocytes were comparable between tumor-bearing and tumor-free animals. This outcome is in line with a study focusing on virus-specific T cell responses using an oncolytic reovirus.^51^ Although pre-existing immunity is not a factor assumed to play a role in potential clinical VSV-GP applications, currently ongoing clinical trials employ a multi-dose regimen with multiple treatment cycles weeks apart.^52^ We found that T cell responses against VSV-GP were not affected by prior VSV-GP application. An additional polyfunctionality assay showed that newly anti-viral CD8^+^ T cells co-express IFN-γ and the degranulation marker CD107a. This co-expression indicates successful IFN-γ-containing vesicle degranulation upon antigen recognition. The absent tumor necrosis factor alpha (TNF-α) levels are in line with previously published data following homologous VSV-GP treatment.^6^ Similarly, other studies showed that TNF-α may not be essential for *in vivo* control of some acute virus infections.^53^^,^^54^ Future studies could assess the phenotype of CD8^+^ T cells derived from these newly identified epitopes.

An indirect way to monitor antigen-specific CD8^+^ T cells is by measuring their IFN-γ production and secretion following *ex vivo* peptide stimulation. However, a faster way is to directly measure antigen-specific CD8^+^ T cells using pMHC-I multimers. Commonly used multimer tools include tetramers, in which the pMHC-I complex is tetramerized on streptavidin together with a fluorophore, and dextramers, which consist of a dextran backbone carrying more fluorophores and pMHCs. These fluorophores coupled to pMHC-I complexes enable a direct visualization using flow cytometry on peripheral blood samples without the need to sacrifice the mouse for splenocyte harvest.^55^ Five custom-made multimers were tested for their ability to identify VSV-GP-specific CD8^+^ T cells. Multimers for H2-Ld-N1 and H2-Ld-N13 clearly labeled CD8^+^ T cells among splenocytes of VSV-GP-immunized mice without a background signal in untreated mice. Additionally, as expected, no multimer signal was detected among CD4^+^ T cells (data not shown). Unexpectedly, the H2-Kd-P16 multimer displayed only a low signal, and H2-Kd-N6 and H2-Kd-P25 showed no statistically significant signal at all, even though all epitopes had been identified and validated with ELISpot and ICS. The discrepancies between these assays might be due to differences in TCR-pMHC multimer interaction kinetics.^56^ Rubio-Godoy et al. demonstrated that the stability of the TCR-pMHC complex affects the efficiency of cell staining with multimers, illustrated in dissociation experiments measuring the multimer’s fluorescence intensity over time. A stable TCR-pMHC complex correlated with efficient multimer staining, while unstable complexes showed a strong decrease in staining ability or failed to stain the target cells at all.^56^ In this context, it is important to mention a drawback of the multimer technology. For a sufficient multimer signal, the affinity of TCR-pMHCs must be higher than the affinity that is needed to trigger T cell activation. This implies that the number of actual antigen-specific CD8^+^ T cells might be underestimated as pMHC multimers might fail to detect functional CD8^+^ T cells.^57^ In addition, optimizations in the multimer staining protocol could potentially help to get clearer and better separated pMHC-I multimer^+^ populations. The interaction of pMHC and TCR leads to a rapid internalization of this complex in a protein tyrosine kinase-dependent manner.^58^ Contrary to published work, pre-treatment with the protein kinase inhibitor dasatinib did not improve the staining intensity of specific CD8^+^ T cells (data not shown).^59^ Furthermore, variations in incubation temperature and time conditions or the addition of a fluorochrome-specific antibody that crosslinks and therefore stabilizes the pMHC-I multimers have shown to positively influence the staining performance.^56^^,^^60^

Taken together, using a multi-level adapted bioinformatic epitope prediction approach together with immune assays, we identified previously unknown VSV-GP-specific CD8^+^ T cell epitopes presented by the BALB/c-specific MHC-I alleles H2-Kd, H2-Dd, and H2-Ld. These findings enable the monitoring of anti-viral CD8^+^ T cell dynamic and epitope distribution patterns of new VSV variants and vaccines in the widely used preclinical BALB/c mouse model. This approach can be used for developing similar tools for human HLAs. With the OV VSV-GP having entered clinical phase 1 testing (NCT05155332), having monitoring tools to accurately detect and characterize virus-specific CD8^+^ T cell responses may provide crucial insights into T cell dynamics in patients undergoing virotherapy treatments. This could, in turn, help to comprehensively understand the induced tumor-specific immune responses and may optimize the monitoring of therapeutic efficacy of this oncolytic virotherapy.

## Materials and methods

### Epitope prediction

The workflow of VSV-GP epitope predictions has been described previously for the C57BL/6J mouse model.^17^ In the current study, this workflow was used for the BALB/c mouse model to predict 8-11mer H2-Kd-, H2-Ld-, and H2-Dd-presented epitopes. Briefly, based on the amino acid sequences encoded by the VSV-GP genome,^2^ three different prediction tools (netMHCpan 4.1, MHCflurry 2.0, and netMHCstabPan) were used for the prediction of strong, weak, and marginal binders, as well as to estimate their immunogenicity score. The 50 highest-ranked epitopes per MHC-I allele were included in a number of peptide candidate pools arranged in a matrix format. As the genes of VSV-GP are obligatorily transcribed sequentially, leading to a gradient amount of viral proteins downstream of the 3′ start site,^61^ a decreasing number of peptides per protein were included in each matrix (15 peptides for the VSV-N protein, 15 for the VSV-P protein, 10 for the VSV-M protein, 5 for the LCMV-GP protein, and 5 for the VSV-L protein). These peptides were included in 14 pools per MHC-I allele (seven horizontal and seven vertical pools), each peptide being present in two pools to allow matrix deconvolution.

### Peptides

Fourteen custom-made peptide pools and 50 individual peptide candidates per MHC-I allele were produced by and purchased from JPT Peptide Technologies (Berlin, Germany). They were resuspended in DMSO (Sigma-Aldrich/Merck #2650, Taufkirchen, Germany) and stored at −80°C until further use as described further. The peptide concentrations used are described in the respective experiment.

### Ethical approval and mouse housing

All animal procedures were approved by the Institutional Animal Care and Use Committee (ZVTA) of the Medical University of Innsbruck and the Austrian Federal Ministry of Science, Research and Economy (BMBWF, 2020–0.475.503). Female 6- to 8-week-old BALB/cJRj mice were purchased from Janvier (Le Genest St Isle, France). Mice were housed in cages in a BSL2 facility at 20°C–24°C with a light/dark cycle of 12 h.

### *In vivo* mouse experiments and tissue harvest

The production of VSV-GP and its titration via TCID_50_ assay were performed as previously described.^6^^,^^62^ For tumor experiments, the colon carcinoma cell line CT26.CL25 (ATCC #CRL-2639, Manassas, VA, USA) was used. Cells were cultured in complete RPMI-1640 medium (Sigma-Aldrich #R0883) containing 10% fetal bovine serum (FBS; PAN-Biotech #P30-3306, Aidenbach, Germany), 1× GlutaMAX (Gibco #A12860-01, Carlsbad, CA, USA), 10 mM HEPES (Gibco #15630-056), 1 mM sodium pyruvate (Gibco #11360-039), 0.1 mM non-essential amino acids (NEAAs; Gibco, #11140-050), 100 U/mL penicillin and 100 μg/mL streptomycin (Gibco #15140-122), and 400 μg/mL G418 (InvivoGen #ant-gn-5, Toulouse, France). Tumors were grafted subcutaneously (s.c.) into the right flank using 1 × 10^5^ cells in PBS. The tumor size (formula: 0.4 × length × width^2^) and weight were monitored every 2–3 days. At a tumor size of around 0.1 cm^3^, mice were re-grouped for evenly distributed tumors and either treated i.v. or i.t. with 10^8^ TCID_50_ VSV-GP in PBS. Seven days post treatment, mice were euthanized to dissect spleens and tumors. Spleens were mechanically dissociated using a 40 μm cell strainer to prepare single-cell suspensions. Subsequently, erythrocyte lysis was performed using the BD Pharm Lyse buffer (BD Biosciences #555899, San Jose, CA, USA). Following the manufacturer’s instructions, tumors were digested utilizing the mouse tumor dissociation kit (Miltenyi Biotec #130-096-730, Bergisch Gladbach, Germany) and the gentleMACS Dissociator (Miltenyi Biotec). Then, tumor samples were smashed through a 70 μm cell strainer, and CD45^+^ cells were isolated using the CD45 (TIL) MicroBeads (Miltenyi Biotec #130-110-618). For the ELISpot assay and ICS, cells were resuspended in T cell medium consisting of DMEM high glucose (Sigma-Aldrich #D5671) supplemented with 10% FBS, 100 U/mL penicillin and 100 μg/mL streptomycin, 10 mM HEPES, 0.1 mM NEAAs, 1× GlutaMAX, and 50 μM β-mercaptoethanol (Gibco #31350-010). For the pMHC-multimer staining, cells were resuspended in FACS buffer that consists of PBS with 2% FBS and 2 mM EDTA (Thermo Fisher Scientific #AM9261, Waltham, MA, USA).

### ELISpot assay

The IFN-γ secretion was examined using IFN-γ ELISpot kits (Mabtech, #3321-4APT-10, Cincinnati, OH, USA) following the manufacturer’s instructions. In brief, ELISpot plates were prepared by washing and blocking (blocking media: DMEM high glucose containing 10% FBS), followed by adding diluted peptides and 2.5 × 10^5^ splenocytes in T cell medium. Cells were stimulated with peptides at a concentration of 35 μg/mL for peptide pools or 5 μg/mL for individual peptides. As a negative control, splenocytes were incubated in T cell medium only. Concanavalin A (ConA; Sigma-Aldrich #C0412) at a final concentration of 5 μg/mL was used as a positive control. ELISpot plates were incubated for approximately 18 h at 37°C with 5% CO_2_. Then, cells were removed and plates were washed five times with PBS. Following incubation with the detection antibody (1 μg/mL) in PBS with 0.5% FBS for 2 h, plates were washed again and incubated with streptavidin-ALP for 1 h. After one more washing step, the plates were developed with the BCIP/NBT-plus substrate, which was stopped after 5 min. Plates were scanned and spots were counted with the ImmunoSpot S6 Ultra V Analyzer (CTL, Shaker Heights, OH, USA) using the ImmunoSpot software version 7.0.20.

### ICS and polyfunctionality staining

To determine intracellular IFN-γ production, 7.5 × 10^5^ splenocytes or TILs were stimulated with 10 μg/mL peptides for 5 h at 37°C and 5% CO_2_. Phorbol-12-myristate-13-acetate (Sigma-Aldrich #P8139; 10 μg/mL) together with ionomycin (Sigma-Aldrich #I0634; 1 μg/mL) served as a positive control, and unstimulated cells were used as a negative control. Subsequently, GolgiPlug (BD Biosciences #555029; 1 μL/mL) was added to each sample. No longer than 12 h after the GolgiPlug addition, cells were resuspended in FACS buffer. For detection of polyfunctional CD8^+^ T cells, 1 × 10^6^ cells were stimulated with peptides in the presence of anti-mouse CD107a-BV421 antibody (BioLegend #121618, San Diego, CA, USA) for 7 h together with GolgiPlug. Cell surface staining was performed using the following antibodies: anti-mouse CD3-AF488 (BioLegend #100210, anti-mouse CD45.2-PerCP-Cy5.5 (BioLegend #109828), anti-mouse CD8a-BV421 (BD Biosciences #563898), anti-mouse CD4-BV510 (BioLegend #100449), anti-mouse CD45.2-AF488 (BioLegend #109816), anti-mouse CD90.2-AF700 (BioLegend #105320), anti-mouse CD8a-BV510 (BioLegend #100752), and anti-mouse CD279-PE/Fire700 (BioLegend #135267). Dead cells were identified and excluded with the LIVE/DEAD Fixable Near-IR Dead Cell Stain (Thermo Fisher Scientific #L34976). Cells were stained at 4°C in the dark for 30 min. After washing with FACS buffer twice, cells were fixed and permeabilized in 100 μL Cytofix/Cytoperm (BD Biosciences #554722) for 30 min at 4°C in the dark, followed by two washing steps with perm/wash buffer (BD Biosciences #554723). For intracellular staining, anti-mouse IFN-γ-APC (BioLegend #505810) and anti-mouse TNFα-PE (BioLegend #506306) antibodies or the corresponding isotype controls (IgG1-APC, BioLegend #400411; IgG1-PE, BioLegend #400408) were diluted in perm/wash buffer and added to the cells for 30 min at 4°C in the dark. After washing, cells were resuspended in FACS buffer, acquired on an FACS Canto II (BD Biosciences) or MACSQuant Analyzer 16 (Miltenyi Biotec) and analyzed using the FlowJo software (version 10.9.0, BD Biosciences). Total cell counts were calculated using Precision count beads (BioLegend #424902).

### pMHC-I multimer staining

For the detection of virus-specific CD8^+^ T cells without *ex vivo* peptide stimulation, five ELISpot- and ICS-validated epitopes were selected to generate custom pMHC-I multimers. One custom tetramer (H2-Ld-N13 YMPYLIDFGL-PE; MBL, Des Plaines, IL, USA) and four custom dextramers (H2-Ld-N1 MPYLIDFGL-PE, H2-Kd-N6 FHFWGQLTAL-PE, H2-Kd-P16 EYLKSYSRL-PE, and H2-Kd-P25 REYLKSYSRL-PE; Immudex, Virum, Denmark) were purchased and tested in an immunization experiment. The immunization of BALB/c mice with 10^8^ TCID_50_ VSV-GP in PBS was performed as described earlier, as well as the splenocyte isolation 7 days later and the preparation of the single-cell suspension. For each staining, 10^6^ splenocytes were incubated with the respective multimer at room temperature for 20 min in the dark. Afterward, cells were incubated with the following staining mix for 30 min at 4°C in the dark: anti-mouse CD45.2-BV421 (BD Biosciences #109832), anti-mouse CD3-PE-Cy7 (BD Biosciences #560591), and anti-mouse CD8a-BV510 (BioLegend #100752). LIVE/DEAD Fixable Near-IR Dead Cell Stain and the dump-channel exclusion markers anti-mouse CD4-APC-Cy7 (BioLegend #100414), anti-mouse CD19-APC-Cy7 (BioLegend #115530), and anti-mouse CD14-APC-Cy7 (BioLegend #123318) were used. After two washing steps with FACS buffer, samples were acquired on an FACS Canto II and analyzed using the FlowJo software.

### Data analysis and statistics

Data were analyzed and visualized with the GraphPad Prism software version 9 (GraphPad Software, La Jolla, CA, US). The statistical tests that were used are indicated in the respective figure legends. A *p* value of <0.05 was considered statistically significant, and differences are indicated with asterisks (∗*p* < 0.05; ∗∗*p* ≤ 0.01; ∗∗∗*p* ≤ 0.001; ∗∗∗∗*p* ≤ 0.0001).

## Data and code availability

All pertinent data generated in this study are included in the article and the supplemental information. Additional and raw data are available upon request from the corresponding author.

## Acknowledgments

The authors thank all members of the CD-VIT lab for the technical support and the critical feedback on the manuscript. This project was funded by the 10.13039/501100006012Christian Doppler Research Association and Boehringer Ingelheim GmbH & Co. KG and the 10.13039/501100004955Austrian Research Promotion Agency (FFG Bridge Project #877143).

## Author contributions

G.W. conceptualized the study. G.W., S.D., S.V.V., L. Pipperger, T.H., and K.D. designed the experiments. G.F. and H.H. performed the bioinformatics analyses. S.D., S.V.V., L. Pipperger, L. Perro, and V.K. performed the experiments. S.D., S.V.V., T.H., and L. Pipperger analyzed data. S.D. wrote the original manuscript. G.W., S.V.V., L. Pipperger, K.D., T.H., and H.H. revised the manuscript.

## Declaration of interests

K.D. is an employee of ViraTherapeutics GmbH. G.W. serves as a scientific advisor for and receives research funding from Boehringer Ingelheim GmbH.

## Supplemental information

Document S1. Figures S1–S5 and Table S1

Document S2. Article plus supplemental information
