# Supplementary material for: Newly identified oncolytic VSV-GP-specific CD8+ T cell epitopes for monitoring of anti-viral immune responses in the BALB/c mouse model
Source: Mol Ther Oncol. 2025 Oct 24;33(4):201072. doi: 10.1016/j.omton.2025.201072 (PMC12634848; doi:10.1016/j.omton.2025.201072)
Supplement: Document S1. Figures S1–S5 and Table S1 [file mmc1.pdf]

OMTON, Volume *33*

Supplemental information

Newly identified oncolytic VSV-GP-specific CD8$^+$ T cell epitopes for monitoring of anti-viral immune responses in the BALB/c mouse model

Sarah Danklmaier, Saskia V. Vijver, Lisa Pipperger, Gabriel Floriani, Lukas Perro, Vanessa Konrad, Tamara Hofer, Hubert Hackl, Krishna Das, and Guido Wollmann

H2-Kd

	POOL 1	POOL 2	POOL 3	POOL 4	POOL 5	POOL 6	POOL 7	
POOL 8	H2-Kd-N1	H2-Kd-N8	H2-Kd-N15	H2-Kd-P22	H2-Kd-P29	H2-Kd-M36	H2-Kd-GP43	
POOL 9	H2-Kd-N2	H2-Kd-N9	H2-Kd-P16	H2-Kd-P23	H2-Kd-P30	H2-Kd-M37	H2-Kd-GP44	
POOL 10	H2-Kd-N3	H2-Kd-N10	H2-Kd-P17	H2-Kd-P24	H2-Kd-M31	H2-Kd-M38	H2-Kd-GP45	
POOL 11	H2-Kd-N4	H2-Kd-N11	H2-Kd-P18	H2-Kd-P25	H2-Kd-M32	H2-Kd-M39	H2-Kd-L46	
POOL 12	H2-Kd-N5	H2-Kd-N12	H2-Kd-P19	H2-Kd-P26	H2-Kd-M33	H2-Kd-M40	H2-Kd-L47	
POOL 13	H2-Kd-N6	H2-Kd-N13	H2-Kd-P20	H2-Kd-P27	H2-Kd-M34	H2-Kd-GP41	H2-Kd-L48	
POOL 14	H2-Kd-N7	H2-Kd-N14	H2-Kd-P21	H2-Kd-P28	H2-Kd-M35	H2-Kd-GP42	H2-Kd-L49	H2-Kd-L50
							H2-Kd-L50	

	POOL 1	POOL 2	POOL 3	POOL 4	POOL 5	POOL 6	POOL 7	
POOL 8	H2-Kd-N1	H2-Kd-N8	H2-Kd-N15	H2-Kd-P22	H2-Kd-P29	H2-Kd-M36	H2-Kd-GP43	
POOL 9	H2-Kd-N2	H2-Kd-N9	H2-Kd-P16	H2-Kd-P23	H2-Kd-P30	H2-Kd-M37	H2-Kd-GP44	
POOL 10	H2-Kd-N3	H2-Kd-N10	H2-Kd-P17	H2-Kd-P24	H2-Kd-M31	H2-Kd-M38	H2-Kd-GP45	
POOL 11	H2-Kd-N4	H2-Kd-N11	H2-Kd-P18	H2-Kd-P25	H2-Kd-M32	H2-Kd-M39	H2-Kd-L46	
POOL 12	H2-Kd-N5	H2-Kd-N12	H2-Kd-P19	H2-Kd-P26	H2-Kd-M33	H2-Kd-M40	H2-Kd-L47	
POOL 13	H2-Kd-N6	H2-Kd-N13	H2-Kd-P20	H2-Kd-P27	H2-Kd-M34	H2-Kd-GP41	H2-Kd-L48	
POOL 14	H2-Kd-N7	H2-Kd-N14	H2-Kd-P21	H2-Kd-P28	H2-Kd-M35	H2-Kd-GP42	H2-Kd-L49	H2-Kd-L50
							H2-Kd-L50	

H2-Dd

	POOL 1	POOL 2	POOL 3	POOL 4	POOL 5	POOL 6	POOL 7	
POOL 8	H2-Dd-N1	H2-Dd-N8	H2-Dd-N15	H2-Dd-P22	H2-Dd-P29	H2-Dd-M36	H2-Dd-GP43	
POOL 9	H2-Dd-N2	H2-Dd-N9	H2-Dd-P16	H2-Dd-P23	H2-Dd-P30	H2-Dd-M37	H2-Dd-GP44	
POOL 10	H2-Dd-N3	H2-Dd-N10	H2-Dd-P17	H2-Dd-P24	H2-Dd-M31	H2-Dd-M38	H2-Dd-GP45	
POOL 11	H2-Dd-N4	H2-Dd-N11	H2-Dd-P18	H2-Dd-P25	H2-Dd-M32	H2-Dd-M39	H2-Dd-L46	
POOL 12	H2-Dd-N5	H2-Dd-N12	H2-Dd-P19	H2-Dd-P26	H2-Dd-M33	H2-Dd-M40	H2-Dd-L47	
POOL 13	H2-Dd-N6	H2-Dd-N13	H2-Dd-P20	H2-Dd-P27	H2-Dd-M34	H2-Dd-GP41	H2-Dd-L48	
POOL 14	H2-Dd-N7	H2-Dd-N14	H2-Dd-P21	H2-Dd-P28	H2-Dd-M35	H2-Dd-GP42	H2-Dd-L49	H2-Dd-L50
							H2-Dd-L50	

	POOL 1	POOL 2	POOL 3	POOL 4	POOL 5	POOL 6	POOL 7	
POOL 8	H2-Dd-N1	H2-Dd-N8	H2-Dd-N15	H2-Dd-P22	H2-Dd-P29	H2-Dd-M36	H2-Dd-GP43	
POOL 9	H2-Dd-N2	H2-Dd-N9	H2-Dd-P16	H2-Dd-P23	H2-Dd-P30	H2-Dd-M37	H2-Dd-GP44	
POOL 10	H2-Dd-N3	H2-Dd-N10	H2-Dd-P17	H2-Dd-P24	H2-Dd-M31	H2-Dd-M38	H2-Dd-GP45	
POOL 11	H2-Dd-N4	H2-Dd-N11	H2-Dd-P18	H2-Dd-P25	H2-Dd-M32	H2-Dd-M39	H2-Dd-L46	
POOL 12	H2-Dd-N5	H2-Dd-N12	H2-Dd-P19	H2-Dd-P26	H2-Dd-M33	H2-Dd-M40	H2-Dd-L47	
POOL 13	H2-Dd-N6	H2-Dd-N13	H2-Dd-P20	H2-Dd-P27	H2-Dd-M34	H2-Dd-GP41	H2-Dd-L48	
POOL 14	H2-Dd-N7	H2-Dd-N14	H2-Dd-P21	H2-Dd-P28	H2-Dd-M35	H2-Dd-GP42	H2-Dd-L49	H2-Dd-L50
							H2-Dd-L50	

H2-Ld

	POOL 1	POOL 2	POOL 3	POOL 4	POOL 5	POOL 6	POOL 7	
POOL 8	H2-Ld-N1	H2-Ld-N8	H2-Ld-N15	H2-Ld-P22	H2-Ld-P29	H2-Ld-M36	H2-Ld-GP43	
POOL 9	H2-Ld-N2	H2-Ld-N9	H2-Ld-P16	H2-Ld-P23	H2-Ld-P30	H2-Ld-M37	H2-Ld-GP44	
POOL 10	H2-Ld-N3	H2-Ld-N10	H2-Ld-P17	H2-Ld-P24	H2-Ld-M31	H2-Ld-M38	H2-Ld-GP45	
POOL 11	H2-Ld-N4	H2-Ld-N11	H2-Ld-P18	H2-Ld-P25	H2-Ld-M32	H2-Ld-M39	H2-Ld-L46	
POOL 12	H2-Ld-N5	H2-Ld-N12	H2-Ld-P19	H2-Ld-P26	H2-Ld-M33	H2-Ld-M40	H2-Ld-L47	
POOL 13	H2-Ld-N6	H2-Ld-N13	H2-Ld-P20	H2-Ld-P27	H2-Ld-M34	H2-Ld-GP41	H2-Ld-L48	
POOL 14	H2-Ld-N7	H2-Ld-N14	H2-Ld-P21	H2-Ld-P28	H2-Ld-M35	H2-Ld-GP42	H2-Ld-L49	H2-Ld-L50
							H2-Ld-L50	

	POOL 1	POOL 2	POOL 3	POOL 4	POOL 5	POOL 6	POOL 7	
POOL 8	H2-Ld-N1	H2-Ld-N8	H2-Ld-N15	H2-Ld-P22	H2-Ld-P29	H2-Ld-M36	H2-Ld-GP43	
POOL 9	H2-Ld-N2	H2-Ld-N9	H2-Ld-P16	H2-Ld-P23	H2-Ld-P30	H2-Ld-M37	H2-Ld-GP44	
POOL 10	H2-Ld-N3	H2-Ld-N10	H2-Ld-P17	H2-Ld-P24	H2-Ld-M31	H2-Ld-M38	H2-Ld-GP45	
POOL 11	H2-Ld-N4	H2-Ld-N11	H2-Ld-P18	H2-Ld-P25	H2-Ld-M32	H2-Ld-M39	H2-Ld-L46	
POOL 12	H2-Ld-N5	H2-Ld-N12	H2-Ld-P19	H2-Ld-P26	H2-Ld-M33	H2-Ld-M40	H2-Ld-L47	
POOL 13	H2-Ld-N6	H2-Ld-N13	H2-Ld-P20	H2-Ld-P27	H2-Ld-M34	H2-Ld-GP41	H2-Ld-L48	
POOL 14	H2-Ld-N7	H2-Ld-N14	H2-Ld-P21	H2-Ld-P28	H2-Ld-M35	H2-Ld-GP42	H2-Ld-L49	H2-Ld-L50
							H2-Ld-L50	

Figure S1: Matrix deconvolution of H2-Kd, H2-Dd, and H2-Ld peptide pools to identify individual peptide candidates. Red frames indicate statistically significant activating pools after the pool screening. Peptides of non-activating pools were crossed out (indicated by grey colors). Only peptide candidates of statistically significant activating pools were further included in the individual peptide candidate testing. Identified H2-Kd-, H2-Dd-, and H2-Ld-restricted activating peptide candidates are highlighted by green frames.

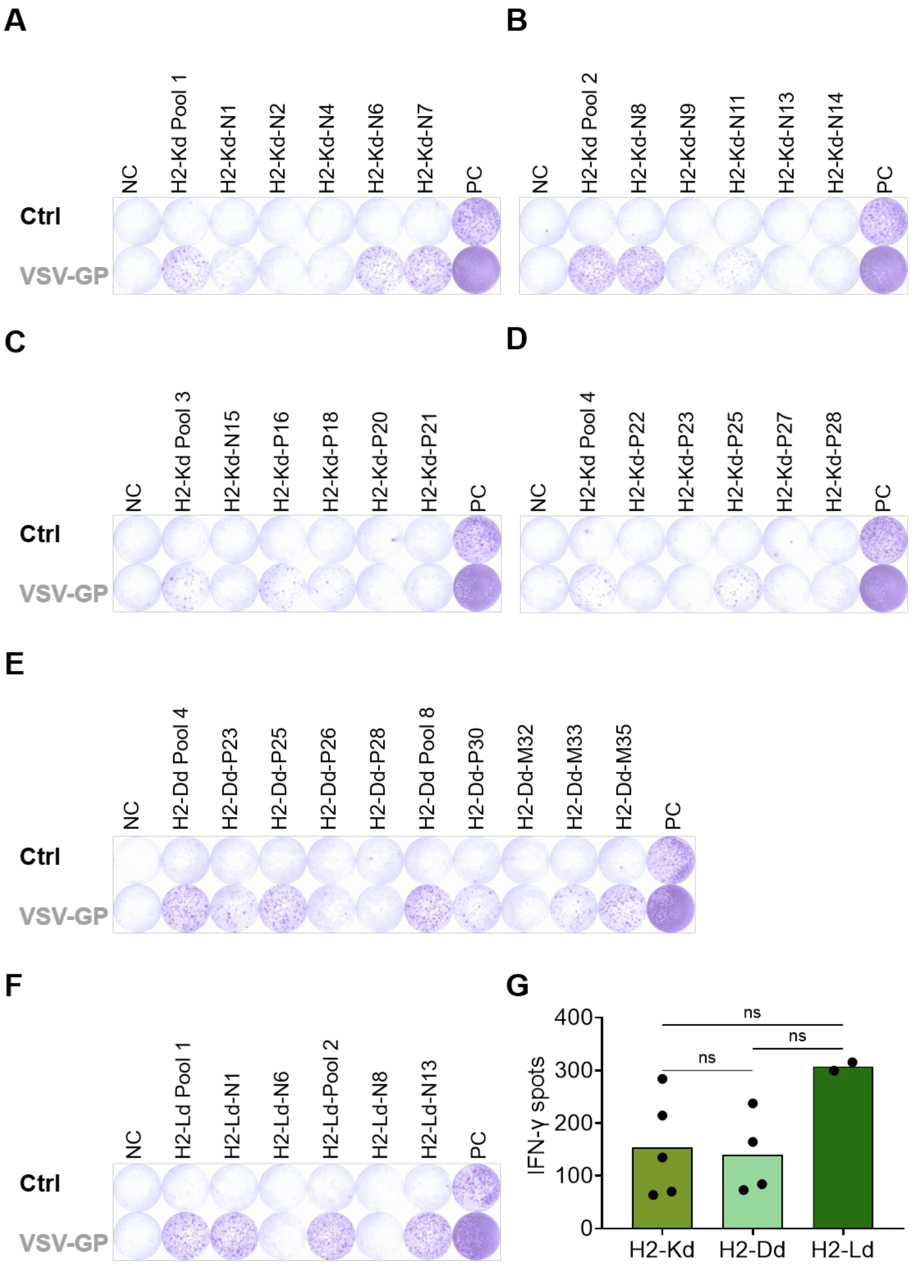

Figure S2: Representative IFN-γ ELISpot images of individual VSV-GP-specific peptide candidate stimulation and distribution of spot counts per MHC-I allele. VSV-GP (10^8 $TCID_{50}$) was injected i.v. into BALB/c mice. Seven days later, spleens of untreated (Ctrl) and VSV-GP-treated (VSV-GP) mice were stimulated with peptides and analyzed in IFN-γ ELISpot assays. Representative IFN-γ ELISpot images of H2-Kd- **(A-D)**, H2-Dd- **(E),** and H2-Ld- **(F)** peptide stimulation and their respective vertical pools are shown. A concentration of 35 µg/ml peptide pool and 5 µg/ml individual peptide was used for stimulation. Unstimulated and ConA-stimulated cells (5 µg/ml) were used as negative control (NC) and positive control (PC), respectively. **(G)** Mean values of IFN-γ spot counts of identified epitopes per MHC allele for H2-

Kd, H2-Dd, and H2-Ld are depicted. Spot counts per MHC-I allele were statistically compared using two-way ANOVA with Tukey's multiple comparison (ns = not significant).

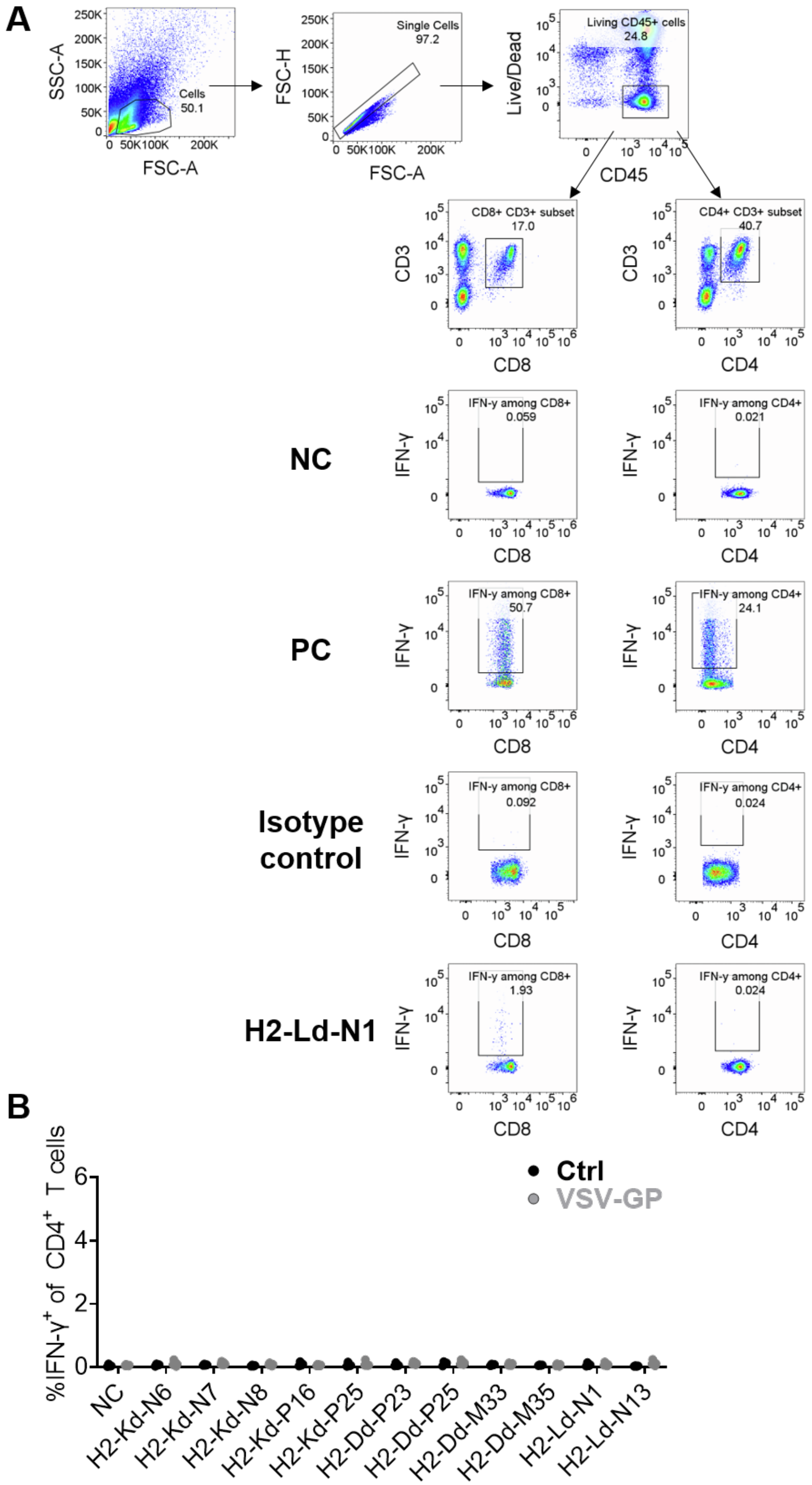
A
SSC-A
FSC-A
Cells
50.1
FSC-H
Single Cells
97.2
Live/Dead
Living CD45+ cells
24.8
CD45
CD8+ CD3+ subset
17.0
CD4+ CD3+ subset
40.7
CD3
CD8
CD4
NC
IFN-γ among CD8+
0.059
IFN-γ among CD4+
0.021
PC
IFN-γ among CD8+
50.7
IFN-γ among CD4+
24.1
Isotype control
IFN-γ among CD8+
0.092
IFN-γ among CD4+
0.024
H2-Ld-N1
IFN-γ among CD8+
1.93
IFN-γ among CD4+
0.024
IFN-γ
B
%IFN-γ+ of CD4+ T cells
Ctrl
VSV-GP
NC
H2-Kd-N6
H2-Kd-N7
H2-Kd-N8
H2-Kd-P16
H2-Kd-P25
H2-Dd-P23
H2-Dd-P25
H2-Dd-M33
H2-Dd-M35
H2-Ld-N1
H2-Ld-N13

Figure S3: Representative ICS gating strategy for IFN-γ^{+} CD8^{+} T and CD4^{+} T cells upon stimulation with VSV-GP-specific peptides. BALB/c mice were treated i.v. with 10^8 $TCID_{50}$ VSV-GP. Seven days later, splenocytes of untreated (Ctrl) and VSV-GP-treated (VSV-GP) mice were analyzed by intracellular IFN-γ staining and measured using flow cytometry. **(A)** A representative gating strategy for the detection of IFN-γ^{+} cells among CD8^{+} and CD4^{+} T cells of a negative control (NC), PMA-ionomycin-stimulated positive control (PC), isotype control, and a VSV-GP-specific H2-Ld-N1 peptide stimulated sample is depicted. **(B)** Frequencies of IFN-γ^{+} cells among CD4^{+} T cells following ICS staining are shown. Unstimulated cells were used as NC. Data are derived from two independent experiments (n = 4 for untreated, n = 6 for VSV-GP). Intracellular IFN-γ production of VSV-GP-immunized samples is compared to untreated mice (unpaired *t*-test) but no statistically significant differences were observed.

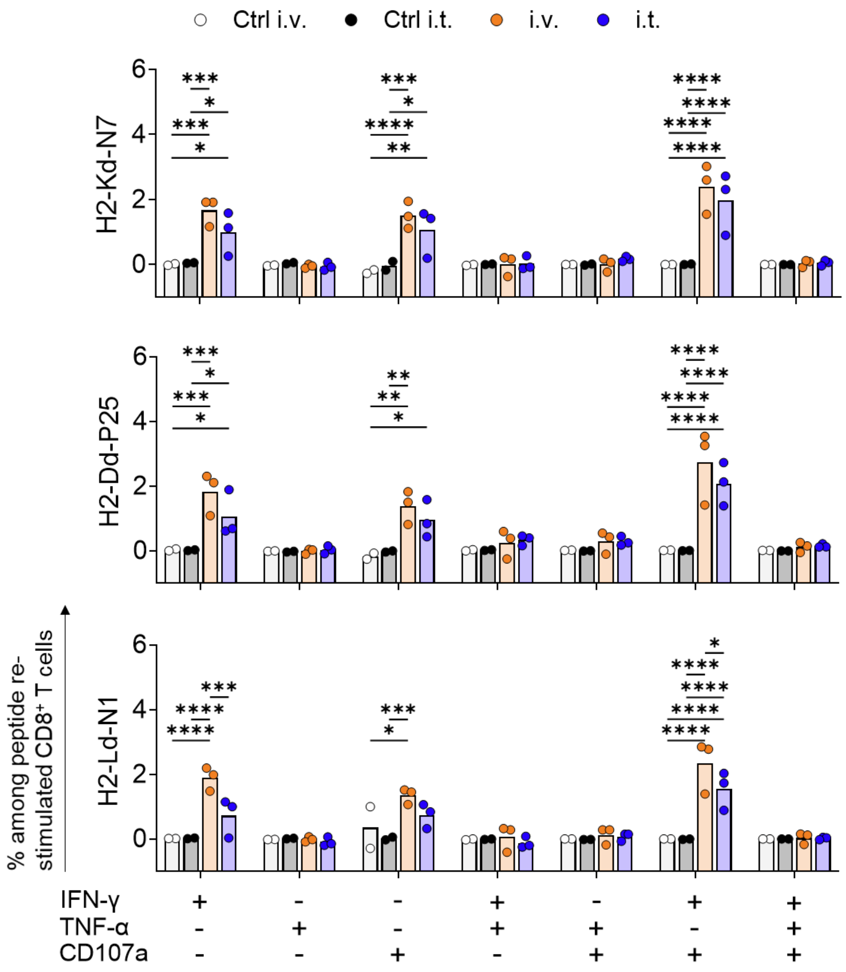

Figure S4: Functionality of VSV-GP-specific CD8$^+$ T cells in the spleen. Mice were treated as depicted in Figure 5. Splenocytes were stained with intracellular IFN-γ, TNF-α, and CD107a antibodies after restimulation (10 µg/ml) with H2-Kd-N7, H2-Dd-P25, and H2-Ld-N1 peptides. Percentages of IFN-γ, TNF-α, and CD107a single positive, double positive and triple positive cells are shown. This study was performed once (n= 2 for untreated and n =3 for VSV-GP). Statistically significant differences are indicated with asterisks (two-way ANOVA with Tukey's multiple comparison). *$p < 0.05$; **$p \leq 0.01$; ***$p \leq 0.001$; ****$p \leq 0.0001$.

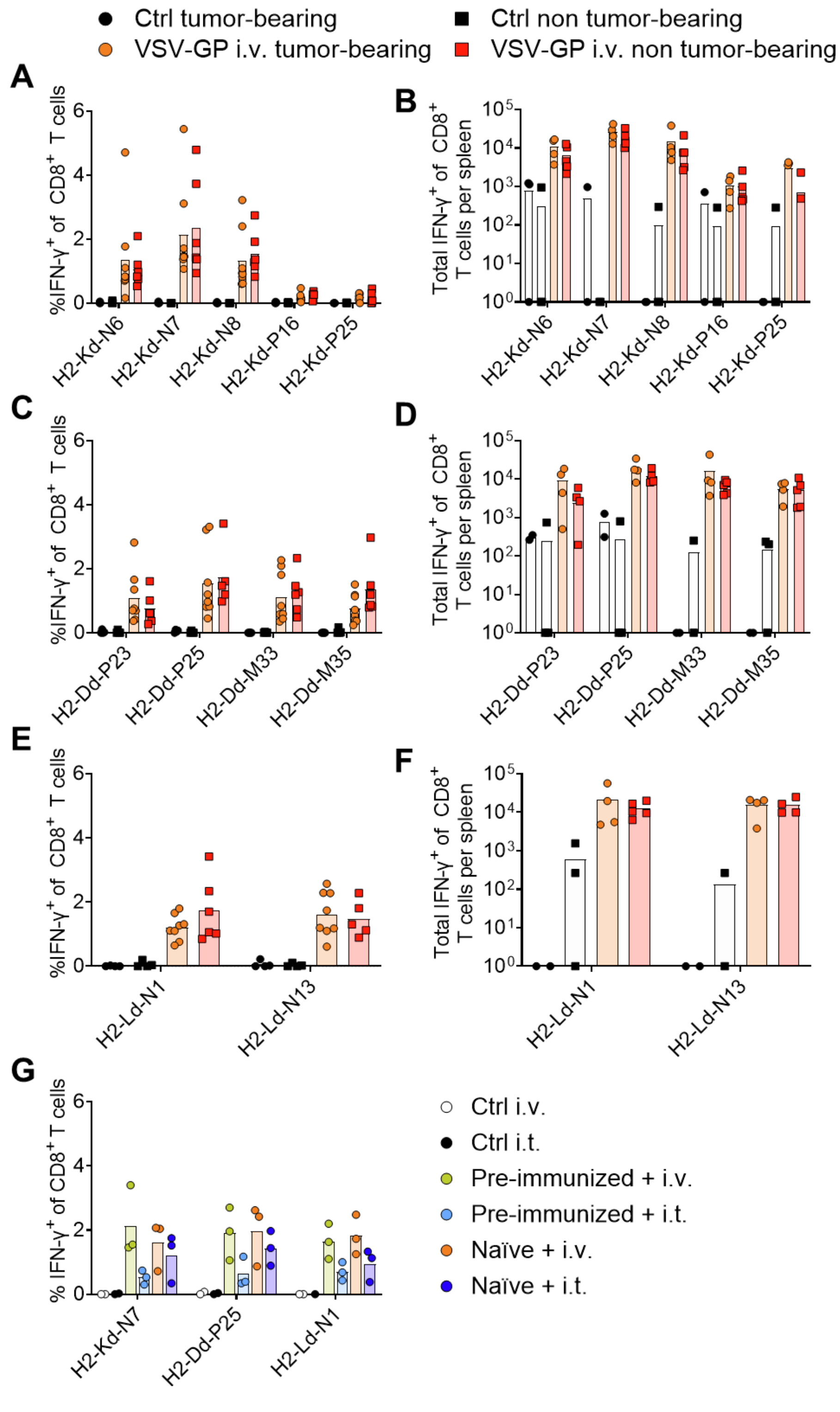

Figure S5: IFN-γ^{+} CD8^{+} T cells upon stimulation with VSV-GP-specific peptides in non-tumor-bearing, tumor-bearing mice and in pre-immunized mice. (A-F) CT26.CL25 tumor-bearing and non-tumor bearing BALB/c mice were immunized i.v. with 10^8 $TCID_{50}$ VSV-GP. Seven days later splenocytes of untreated (Ctrl) and VSV-GP-treated (VSV-GP) animals were

used for IFN-γ ICS staining followed by flow cytometry analysis. **(G)** Mice were additionally pre-immunized i.v. with 10^8 $TCID_{50}$ VSV-GP three weeks prior to the tumor implantation and were compared to non-pre-immunized mice. Proportion **(A, C, E, G)** and absolute counts (**B, D, F**) of IFN-γ^+ $CD8^+$ T cells in response to H2-Kd-, H2-Dd- and H2-Ld-presented VSV-GP-specific peptide stimulation (10 µg/ml) is depicted. **(D)** Three different epitopes were used for peptide restimulation **(A-F)** Data from two independent experiments are displayed (n = 4 for untreated and n = 6-8 for VSV-GP), quantitative analysis was done for one of the experiments. **(G)** Data from one experiment are displayed (n = 2 for untreated and n = 3 for VSV-GP). IFN-γ production of VSV-GP treated tumor-bearing vs non-tumor-bearing, and pre-immunized vs non-pre-immunized mice is compared (two-way ANOVA with Tukey's multiple comparison), but no statistically significant differences were observed.

Table S1: Characteristics of identified BALB/c-restricted VSV-GP-specific CD8⁺ T cell epitopes

Name	MHC-I allele	Protein	Peptide sequence	Amino Acid position	Peptide length	Affinity (nM) *
H2-Ld-N1	Ld	N	MPYLIDFGL	275 - 283	9	23.12
H2-Ld-N13	Ld	N	YMPYLIDFGL	274 - 283	10	786.68
H2-Kd-N6	Kd	N	FHFWGQLTAL	298 - 306	10	951.92
H2-Kd-N7	Kd	N	QYAKRAVMSL	395 - 404	10	>1000
H2-Kd-N8	Kd	N	SYMPYLIDFGL	273 - 283	11	>1000
H2-Kd-P16	Kd	P	EYLKSYSRL	431 - 439	9	48.83
H2-Kd-P25	Kd	P	REYLKSYSRL	430 - 439	10	291.94
H2-Dd-P23	Dd	P	FQPKKASLQPL	629 - 639	11	>1000
H2-Dd-P25	Dd	P	RAEKSNYEL	453 - 461	9	>1000
H2-Dd-M33	Dd	M	SSLKKILGL	689 - 697	9	>1000
H2-Dd-M35	Dd	M	SGAWVLDSI	904 - 912	9	>1000

*NetMHC-4.0 server predicted the peptide-MHC-I binding affinity in nanomolar units. Based on the manufacture's requirements, only peptides with affinity values <1000 nM were selected for peptide-MHC-I multimer generation.
